# Who and what retrospective risk assessments miss: Examining retrospective denial of momentary suicidal ideation in adolescents

**DOI:** 10.1002/jcv2.70063

**Published:** 2025-11-25

**Authors:** Ki Eun Shin, Ilana Gratch, Christine B. Cha

**Affiliations:** ^1^ Yale Child Study Center Yale School of Medicine New Haven Connecticut USA; ^2^ Department of Psychiatry Weill Cornell Medicine New York New York USA

**Keywords:** adolescent, ecological momentary assessment, method variance, retrospective bias, suicide risk assessment

## Abstract

**Background:**

Emerging evidence indicates that real‐time assessments such as ecological momentary assessment (EMA) detect individuals experiencing suicidal ideation (SI) who go undetected by retrospective assessments. However, it remains unclear for whom and why such discrepancies occur. Few prior studies accounted for method variance (e.g., survey vs. interview), and no studies have comprehensively examined which individual characteristics differentiate those with and without discrepant reports across EMA and retrospective measures. The current study aimed to address these gaps by examining discrepancies in SI endorsement across EMA and two forms of retrospective measures (survey, interview), as well as comparing those with and without discrepant reports on demographic, clinical, and EMA‐based variables.

**Methods:**

Eighty community‐based adolescents completed a baseline online survey assessing demographic and clinical characteristics, followed by a 14‐day EMA assessing momentary SI (5x/day). At the conclusion of the EMA period, adolescents retrospectively reported their SI during the past 2 weeks via a survey and an interview.

**Results:**

Of 48 adolescents who endorsed some SI during EMA, 35%–41% retrospectively denied having experienced SI during the EMA period on the follow‐up survey and interview. Adolescents with retrospective denial had a lower likelihood of lifetime SI (*ps* < 0.05, *φ*_*c*_ = 0.53–0.55), lower SI and depression severity (*ps* < 0.05, *ds* = 0.52–0.64), and lower hopelessness at baseline (*ps* < 0.05, *ds* = 0.56–0.60), as well as less frequent and less severe SI (*ps* < 0.05, *ds* = 0.32–0.72) during EMA compared to those who endorsed SI across both EMA and retrospective measures.

**Conclusion:**

Real‐time monitoring detects more adolescents experiencing SI than retrospective assessments, particularly those with lower clinical severity. Therefore, real‐time monitoring has the potential to facilitate early interventions by identifying adolescents who are not yet as advanced in the suicide risk trajectory but are at risk of further escalation.

## Key Points

**What's known?**

- A subset of suicidal individuals deny having experienced suicidal ideation (SI) that they previously reported in real‐time when their suicidal thoughts are assessed retrospectively later.

**What's new?**

- What differentiated between individuals who retrospectively denied their SI and those who did not was lower clinical severity in the former group (e.g., less likely to have experienced SI before, lower SI and depression severity and hopelessness). The former group also experienced less frequent and less severe SI as assessed in real time than the latter group.

**What's relevant?**

- Findings indicate the benefits of real‐time monitoring relative to retrospective assessments in detecting more adolescents experiencing SI, particularly those who currently present with lower clinical severity.

## INTRODUCTION

Adolescence represents a relatively high‐risk period for the emergence of suicidal thoughts and behaviors (STBs), with 17% of U.S. high school students reporting seriously considering suicide in the past year (Kann, 2018). Detecting STBs through assessment affords an opportunity to intervene and, ideally, prevent them from escalating further. Assessment of adolescents' STBs, in both research and clinical practice, typically hinges upon adolescents' reports elicited via retrospective surveys (e.g., the Suicidal Ideation Questionnaire; Reynolds, 1987) and retrospective interviews (e.g., the Columbia Suicide Severity Rating Scale; Posner et al., 2011). However, assessments relying on retrospective recall may have limited sensitivity given that suicidal thoughts tend to be transient and rapidly fluctuating (e.g., over a few hours; Kleiman et al., 2017). In light of this limitation, there has been increasing interest in expanding suicide risk assessments to include momentary reports made by people in real‐time through ecological momentary assessment (EMA), which involves multiple brief surveys administered over a discrete period of time (Kivelä et al., 2022; Sedano‐Capdevila et al., 2021).

A growing body of research has focused on comparing the use of retrospective measures and EMA with regard to assessment of STBs (e.g., Forkmann et al., 2018; Gratch, Choo, et al., 2021; Czyz et al., 2018). Most of these studies found that patients were more likely to report experiencing suicidal ideation (SI) in real‐time via EMA than retrospectively at the end of the assessment period (e.g., Czyz et al., 2018; Forkmann et al., 2018; Gratch, Choo, et al., 2021). Among young people specifically, Czyz et al. (2018) found 70.6% of recently discharged adolescents reported SI via daily diary over 1 month versus only 45.2% via retrospective report at the month's end. Similarly, Esposito et al. (2022) found that of 30 adolescents reporting suicidality following acute psychiatric care, 100% of them reported ideation via EMA, while only 57% did so in a retrospective interview administered over the phone. While the prior studies have begun to help clarify that there is some degree of discrepancy across retrospective and EMA‐based methods of assessing STBs, there remain several gaps in the literature. First, only a handful of studies have focused on adolescents (i.e., Czyz et al., 2018; Esposito et al., 2022). Given that STBs tend to onset during adolescence (Nock et al., 2008, 2013), when one's understanding of the construct of suicide/death is still maturing (Honey & Dark‐Freudeman, 2024; Noppe & Noppe, 1997; Tezanos et al., 2024), developmental factors may play a role in the nature and extent of discrepant reporting. Second, those few studies examining youth have focused exclusively on adolescents already engaged in some form of psychiatric treatment (Czyz et al., 2018; Esposito et al., 2022). Given the low rates of treatment‐seeking among suicidal adolescents (<30%; Hom et al., 2015), and the importance of improving our ability to detect STBs among those who have not yet interfaced with the mental healthcare system, it is important to extend the prior studies by sampling from community‐based adolescents who represent a wider range of STB severity and treatment‐seeking history.

A further limitation is that most prior studies (e.g., Esposito et al., 2022; Gratch, Choo, et al., 2021) compared EMA to retrospective *interviews*, thus prohibiting conclusions about whether discrepancies were due to difference in timescale of assessment (e.g., right now vs. past 2 weeks), method of delivery (e.g., spoken to another person vs. submitted via survey), or some combination of both. A more direct comparison—for instance, EMA versus retrospective self‐report survey—would help parse these respective pieces apart. Relatedly, some previous studies (e.g., Forkmann et al., 2018) used EMA items that are conceptually similar but not identical in wording to those used in the retrospective measure, introducing the possibility of method variance. Further research accounting for these factors is needed to clarify the source(s) of these discrepancies.

Our understanding about these discrepancies may be further strengthened by incorporating perspectives from social and cognitive psychology, which cast doubt on the extent to which we might expect retrospective and momentary assessments to converge in the first place. For example, Fredrickson and Kahneman's (1993) research on the recollection and reporting of physical pain led to the articulation of the “peak‐end rule,” stating that people judge experiences based primarily on how they felt at the peak point (i.e., most pain) and at the end point. There is preliminary empirical support for this notion in STB research; Gratch, Choo, et al. (2021) found depressed adults' EMA ratings at their “worst point,” or their highest level of SI reported across the EMA period, correlated with their retrospective ratings, but more research is needed to examine the extent to which this principle applies to suicide risk assessments.

Finally, there have been minimal efforts to examine exactly which individuals are more prone to demonstrating the discrepancy between EMA and retrospective assessment of STB. Previous research (e.g., Esposito et al., 2022; Gratch, Choo, et al., 2021; Spears et al., 2022) has emphasized the importance of identifying such correlates in service of better understanding implications for clinical care. Clinical severity at baseline may be one such factor; Gratch, Choo, et al. (2021) found that those who endorsed ideation in real‐time and retrospectively reported more severe depression and hopelessness at baseline than did those who endorsed ideation in real‐time but denied it retrospectively. Demographic factors, which have been linked to varying levels of STB disclosure in other assessment contexts (e.g., greater discrepancies in reports of SI among racial/ethnic minority parent‐adolescent dyads vs. white dyads; Bell et al., 2022), also warrant further examination in this context. Other possible moderators may be factors thought to be linked to disclosure of STBs more broadly, such as stigma, insight, and treatment engagement (Ammerman et al., 2022; Gratch, Choo, et al., 2021; Hom et al., 2017; Spears et al., 2022).

In the current study, we examined discrepancies between EMA and retrospective reports on SI provided by adolescents from the community. To address the aforementioned limitations in the extant literature, we pursued the following specific aims. First, we compared the rate at which community‐based adolescents endorsed SI as measured by 2‐week EMA versus two types of retrospective, aggregate measures (self‐report survey and interview). Second, we compared adolescents with and without discrepant reports across EMA and retrospective measures based on their SI frequency, as well as mean, worst‐point (“peak”), and most recent (“end”) SI intensities during EMA. Finally, we examined potential correlates of the discrepancy between EMA and retrospective measures, including: clinical severity at baseline, demographic factors, mental health stigma, self‐reflection/insight, and treatment engagement during EMA, in addition to participants' perceived accuracy of their responses on retrospective measures.

## METHOD

### Participants

Eighty community‐based adolescents with a mean age of 15.8 years (SD = 1.28) were recruited via online social media advertisements, which featured an array of messages, some referencing suicide/self‐harm and others referencing teen well‐being more broadly. Inclusion criteria included that adolescents were 12–17 years old, resided in the greater New York metropolitan area, endorsed either recent SI (i.e., in the past 2 weeks) or no lifetime history of SI, had access to a smartphone that they could use to complete EMA surveys, and were capable of providing informed assent and attaining parent/guardian consent. Adolescents were excluded if they did not have access to a smartphone, presented with high/imminent suicide risk at the time of phone screening, or were deemed incapable of providing informed consent/assent (e.g., lack of English proficiency, gross cognitive impairment). Given the scope of our investigation on discrepant reports of SI, here we focus on a subsample of 48 adolescents who endorsed at least some SI during the 14‐day EMA period and completed post‐EMA follow‐up assessments. This final sample of 48 included 37 adolescents with recent SI and 11 with no lifetime history of SI. In terms of demographics, most adolescents identified as females based on biological sex (87.50%) and gender (75.00%). Half of the sample identified as sexually minoritized (50.00%), and more than half identified as racially minoritized (56.25%). A small subset (10.42%) identified as Hispanic/Latino (see Table 1).

**TABLE 1:** Comparisons of adolescents with and without EMA‐retrospective report discrepancies on demographic and clinical characteristics.

	Total (*n* = 48)	EMA versus follow‐up survey		*p*	ES	EMA versus follow‐up interview		*p*	ES
		EMA SI+/FU survey SI− (*n* = 20)	EMA SI+/FU survey SI+ (*n* = 28)			EMA SI+/FU interview SI− (*n* = 17)	EMA SI+/FU interview SI+ (*n* = 31)		
Biological sex				1.00	0.06			1.00	0.02
Female	42 (87.50%)	24 (85.71%)	18 (90.00%)			15 (88.24%)	27 (87.10%)		
Male	6 (12.50%)	4 (14.29%)	2 (10.00%)			2 (11.76%)	4 (12.90%)		
Gender				0.573	0.21			0.778	0.15
Female	36 (75.00%)	17 (85.00%)	19 (67.86%)			14 (82.35%)	22 (70.97%)		
Male	6 (12.50%)	2 (10.00%)	4 (14.29%)			2 (11.76%)	4 (12.90%)		
Non‐binary	6 (12.50%)	1 (5.00%)	5 (17.86%)			1 (5.88%)	5 (16.13%)		
Race				0.573	0.31			0.538	0.33
Asian	14 (29.17%)	6 (30.00%)	8 (28.57%)			6 (35.29%)	8 (25.81%)		
Black	7 (14.58%)	5 (25.00%)	2 (7.14%)			4 (23.53%)	3 (9.68%)		
White	21 (43.75%)	8 (40.00%)	13 (46.43%)			7 (41.18%)	14 (45.16%)		
Multiracial	4 (8.33%)	1 (5.00%)	3 (10.71%)			0 (0.00%)	4 (12.90%)		
Other	2 (4.17%)	0 (0.00%)	2 (7.14%)			0 (0.00%)	2 (6.45%)		
Ethnicity				1.00	0.01			0.753	0.11
Hispanic/Latino	5 (10.42%)	2 (10.00%)	3 (10.71%)			1 (5.88%)	4 (12.90%)		
Non‐Hispanic/Latino	43 (89.58%)	18 (90.00%)	25 (89.29%)			16 (94.12%)	27 (87.10%)		
Sexual orientation				0.228	0.36			0.663	0.25
Heterosexual	24 (50.00%)	14 (70.00%)	10 (35.71%)			11 (64.71%)	13 (41.94%)		
Gay/Lesbian	5 (10.42%)	2 (10.00%)	3 (10.71%)			2 (11.76%)	3 (9.68%)		
Bisexual	10 (20.83%)	2 (10.00%)	8 (28.57%)			2 (11.76%)	8 (25.81%)		
Other (e.g., questioning)	9 (18.75%)	2 (10.00%)	7 (25.00%)			2 (11.76%)	7 (22.58%)		
Lifetime suicidal ideation history	37 (77.08%)	10 (50.00%)	27 (96.43%)	0.001*	0.55	8 (47.06%)	29 (93.55%)	0.004*	0.53
Lifetime suicide attempt history	15 (31.25%)	2 (10.00%)	13 (46.43%)	0.037*	0.39	2 (11.76%)	13 (41.94%)	0.130	0.31
Mental health treatment at baseline	18 (37.50%)	3 (15.00%)	15 (53.57%)	0.032*	0.39	3 (17.65%)	15 (48.39%)	0.133	0.30
Started mental health treatment during EMA	1 (2.08%)	1 (5.00%)	0 (0.00%)	0.573	0.17	1 (5.88%)	0 (0.00%)	0.538	0.20
Stopped mental health treatment during EMA	1 (2.08%)	0 (0.00%)	1 (3.57%)	1.00	0.12	0 (0.00%)	1 (3.23%)	1.00	0.11
Perceived accuracy of completing follow‐up measures^a^				0.687	0.09			0.100	0.29
Accurate and easy to complete	36 (83.72%)	16 (80.00%)	20 (87.00%)			19 (95.00%)	17 (73.91%)		
Accurate, but difficult to choose one representative number for ratings	7 (16.28%)	4 (20.00%)	3 (13.00%)			1 (5.00%)	6 (26.09%)		
Age	15.78 (1.28)	15.80 (1.47)	15.17 (1.21)	0.976	0.07	15.59 (1.62)	15.84 (1.13)	0.663	0.09
Suicidal Ideation Questionnaire	41.54 (36.33)	18.45 (23.67)	58.04 (35.02)	0.001*	0.64	18.94 (24.48)	53.94 (36.06)	0.004*	0.52
Quick Inventory of Depression Severity	11.06 (5.49)	7.80 (4.57)	13.39 (4.93)	0.001*	0.58	7.59 (4.72)	12.97 (4.98)	0.004*	0.53
Screen for Child Anxiety Related Disorders	38.27 (15.22)	33.10 (16.69)	41.96 (13.16)	0.125	0.30	31.59 (17.07)	41.94 (12.96)	0.077	0.34
Ruminative Response Scale	24.69 (7.11)	22.25 (7.36)	26.43 (6.51)	0.114	0.30	22.00 (8.27)	26.16 (6.04)	0.130	0.29
Penn State Worry Questionnaire for Children	26.15 (9.36)	24.20 (10.40)	26.15 (9.36)	0.383	0.18	23.24 (10.11)	27.74 (8.68)	0.224	0.24
Hopelessness Scale for Children	5.69 (3.45)	3.60 (2.39)	7.18 (3.35)	0.001*	0.60	3.41 (2.45)	6.94 (3.31)	0.004*	0.56
Stigma‐9 Questionnaire	15.31 (6.26)	13.95 (7.01)	16.29 (5.59)	0.382	0.19	13.71 (6.26)	16.19 (6.18)	0.347	0.19
Self‐Reflection and Insight Scale for Youth, Self‐Reflection Subscale	50.50 (10.00)	49.65 (9.79)	51.11 (10.27)	0.775	0.07	49.24 (10.33)	51.19 (9.91)	0.663	0.09
Self‐Reflection and Insight Scale for Youth, Insight Subscale	18.33 (6.43)	20.20 (7.46)	5.33 (17.00)	0.200	0.25	21.35 (8.13)	16.67 (4.64)	0.056	0.37

*Note*: Frequency and percentage are reported for categorical variables, and mean and standard deviation are reported for continuous variables. Effect size represents Cramer's *V* for categorical variables and Cohen's *d* for continuous variables. Reported *p*‐values were adjusted using the Benjamini‐Hochberg Procedure to control the false discovery rate.

Abbreviations: EMA, ecological momentary assessment; ES, effect size.

^a^ Due to 5 participants not providing responses on the exit survey, results are reported based on *n* = 43.

*p < 0.05; **p < 0.001.

### Procedure

Recruited adolescents and their parents/guardians met with research team members via a HIPAA‐compliant video‐conferencing platform to complete online consent/assent forms. Then, adolescents completed an online survey for the baseline assessment and received an orientation to EMA. They learned about the EMA survey delivery schedules and completed a practice EMA survey in the Metricwire app. For the following 14‐day period, adolescents were sent an EMA survey five times daily. On weekdays, adolescents received the first survey of the day at their designated typical wake time. The following three surveys were administered randomly within fixed intervals outside of school hours, and the final survey was sent at 10 pm each evening. Weekend schedules were similar except that the middle three surveys were spread across the day (vs. post‐school hours). Response window for EMA surveys ranged between 30 and 60 min. EMA items assessed adolescents' momentary future thinking and SI. Research team monitored adolescents' daily endorsement of suicidality and EMA response rates to ensure participant safety and compliance. In case an adolescent indicated elevated ratings of imminent suicidal intent and/or difficulty keeping oneself safe, a licensed clinical psychologist contacted the participant to check in and if risk levels did not mitigate, informed the adolescent's parent/guardian. Within a week from the ending of the EMA period, adolescents had the second video call with the research staff to complete a follow‐up assessment, in which both an online survey and an interview were administered by the research staff. The order of survey and interview administration was counterbalanced across participants. Adolescents also received instruction on uninstalling the EMA app and were debriefed. All participants received gift cards as compensation in the amount up to $100 ($20 for baseline, $14 for follow‐up, and up to $64 for EMA based on the rate of $0.80 per survey in addition to a $8 bonus for 80% or higher compliance). All study procedures were approved by the Teachers College, Columbia University IRB.

### Measures

#### Baseline measures

Baseline online survey assessed participant demographics, including their age, sex, race, ethnicity, gender, and sexual orientation. Participants also reported whether they were receiving any form of mental health treatment at baseline. We also assessed recent history of SI (in the past 2 weeks) using the Suicidal Ideation Questionnaire (SIQ; Reynolds, 1987) and lifetime history using the Self‐Injurious Thoughts and Behaviors Interview‐Revised (SITBI‐R; Fox et al., 2020; Gratch, Fernandes, et al., 2021). Recent history of depressive (in the past week) and anxiety symptoms (in the past 3 months) were assessed using the Quick Inventory for Depression Symptoms (QIDS; Rush et al., 2003) and the Screen for Child Anxiety Related Disorders (SCARED; Birmaher et al., 1999), respectively. Other clinical constructs assessed include rumination based on the Ruminative Response Scale (RRS; Treynor et al., 2003), worry based on the Penn State Worry Questionnaire for Children (PSWQ‐C; Chorpita et al., 1997), and hopelessness based on the Hopelessness Scale for Children (HSC; Kazdin et al., 1986). As potential correlates of retrospective assessment‐EMA discrepancies in the reporting of SI, mental health stigma and self‐reflection and insight were also assessed using the Stigma‐9 Questionnaire (STIG‐9; Gierk et al., 2018) and the Self‐Reflection and Insight Scale for Youth (SRIS‐Y; Sauter et al., 2010). The baseline self‐report measures were previously validated and demonstrated strong psychometric properties, including high internal consistency (Cronbach's *α* = 0.77–0.97 in the current sample).

#### EMA survey

The survey included self‐report items assessing participants' momentary cognitions (e.g., temporal orientation, emotional content) as well as momentary suicide‐related thoughts. Four items were adapted from a prior validation study (Forkmann et al., 2018) to assess momentary SI (“At this moment, or just recently”). Two items assessed passive ideation (“life is/was not worth living for me,” “there are/were more reasons to die than to live for me”), and the other two items assessed active ideation (“I want(ed) to die,” “I think/thought about taking my life”).^1^ SI items were rated on a 7‐point Likert scale (1 = *Not at all*; 7 = *Completely*).^2^ SI composite scores were calculated by averaging across the four SI items such that the score of 1 indicated no SI. Participants were deemed to have endorsed each type of SI during EMA if their ratings on each SI item and SI composite score were greater than 1 on at least one EMA survey across the 2‐week period. The four SI items demonstrated good internal consistency at both within‐ and between‐person levels (within‐person *ω* = 0.84; between‐person *ω* = 0.84). These estimates were comparable to what was observed in the original validation study (within‐person *ω* = 0.79–0.80; between‐person *ω* = 0.92–0.97; Forkmann et al., 2018).

#### Follow‐up measures

Follow‐up assessments involved both online survey and interview conducted by research staff. The survey assessed participants' experiences with EMA in the current study, as well as any change in their mental health treatment status during the EMA period. During the survey, participants provided retrospective, aggregated ratings on their SI for the EMA period based on the four SI items from EMA (e.g., “Over the past 2 weeks, life was not worth living for me”), rated on the same Likert scale (1 = *Not at all;* 7 = *Completely*). During a follow‐up interview, a research team member verbally administered the identical SI items to participants. The item wording and response scales remained consistent with EMA to control for potential method variance beyond the timescale of assessment. SI composite scores were calculated based on the follow‐up survey and interview separately by averaging across individual items. Participants were considered to have endorsed SI if their ratings on each SI item and the SI composite score were greater than 1, as determined by the follow‐up survey and interview, respectively. SI items demonstrated high internal consistency based on both survey (*α* = 0.87) and interview (*α* = 0.85). At the end of the follow‐up survey, participants were provided with a separate link to an exit survey, adapted from a prior study (Deming et al., 2021). The exit survey was intended to be completed immediately following the end of the video call and assessed adolescents' perceived efforts and accuracy in how they responded to suicide‐related items on the follow‐up online survey and interview. To facilitate honest reporting, adolescents were informed that their responses on the exit survey would remain confidential, would not affect their compensation, and would not involve a follow‐up safety measure (e.g., risk assessment).

### Data analysis

Preliminary analyses were conducted to examine average EMA compliance in the sample as well as descriptives on EMA‐based SI (both individual items and the aforementioned SI composite). We examined three types of EMA SI ratings: Within‐person average SI ratings (“mean”), within‐person maximum or highest SI rating during the EMA period (“worst‐point”), and each participant's average SI rating across the final day of their EMA response submission (“end‐point”). The frequency of discrepancies in retrospective self‐reports on SI between the follow‐up survey and interview was also examined.

For primary analyses, descriptive statistics were conducted to examine the frequency of discrepant self‐report on SI between EMA and each of the follow‐up assessments (survey, interview). Once adolescents with discrepant versus consistent reports across EMA and the follow‐up assessments were identified, group comparisons were conducted on EMA SI ratings (SI frequency, mean, worst‐point, and end‐point levels during the 2‐week EMA period) and EMA compliance. To account for skewness in the data (e.g., infrequent endorsement of SI levels >1), comparisons were performed using independent samples *t*‐tests with bias‐corrected bootstrapped *p*‐values based on 10,000 resamples. Groups were also compared on a range of demographic and clinical variables, as well as adolescents' perceived accuracy of their follow‐up assessment responses. Independent samples *t*‐tests and Fisher's exact tests were conducted. For each set of group comparisons, the Benjamini‐Hochberg Procedure (Benjamini & Hochberg, 1995) was used to adjust *p*‐values and control false discovery rates.

## RESULTS

### Preliminary results

On average, adolescents responded to 52.42 EMA surveys (SD = 12.29). This corresponded to 74.89% compliance. On average, adolescents endorsed some SI (i.e., >1 on SI composite) on 14.19 occasions (SD = 14.75, range = 1–55), or 29.74% of total EMA survey submissions (SD = 29.83). During the 14‐day EMA period, adolescents endorsed low levels of SI on average and at the “end‐point” or the last time they reported SI. Adolescents' ratings at their worst point indicated moderate levels of SI, on average (see Table 2).

**TABLE 2:** Comparison of adolescents with and without EMA‐retrospective report discrepancies on EMA‐based suicidal ideation frequency and severity.

		EMA versus follow‐up survey		*p*	*d*	EMA versus follow‐up interview		*p*	*d*
	Total (*n* = 48)	EMA SI+/FU survey SI− (*n* = 20)	EMA SI+/FU survey SI+ (*n* = 28)			EMA SI+/FU interview SI− (*n* = 17)	EMA SI+/FU interview SI+ (*n* = 31)		
	*M* (SD)	*M* (SD)	*M* (SD)			*M* (SD)	*M* (SD)		
EMA SI frequency									
“Life not worth living”	10.25 (12.15)	3.00 (5.66)	15.43 (12.95)	0.002*	0.59	3.47 (6.12)	13.97 (13.07)	0.003*	0.45
“More reasons to die than to live”	8.23 (12.00)	2.25 (5.47)	12.50 (13.57)	0.008*	0.47	2.65 (5.94)	11.29 (13.39)	0.014*	0.37
“Want to die”	10.00 (11.66)	2.40 (5.54)	15.43 (11.91)	<0.001**	0.66	2.59 (6.01)	14.06 (12.07)	0.002*	0.53
“Think about taking life”	5.77 (8.00)	1.80 (4.91)	8.61 (8.63)	0.003*	0.46	2.18 (5.29)	7.74 (8.60)	0.018*	0.35
SI composite	14.19 (14.75)	4.90 (7.23)	20.82 (15.24)	<0.001**	0.63	5.24 (7.80)	19.10 (15.43)	0.002*	0.50
EMA SI mean severity									
“Life not worth living”	1.46 (0.72)	1.09 (0.18)	1.27 (0.84)	0.012*	0.48	1.12 (0.21)	1.64 (0.83)	0.025*	0.37
“More reasons to die than to live”	1.38 (0.71)	1.07 (0.17)	1.60 (0.86)	0.003*	0.40	1.09 (0.19)	1.54 (0.84)	0.047*	0.32
“Want to die”	1.44 (0.65)	1.07 (0.19)	1.71 (0.74)	0.006*	0.55	1.09 (0.21)	1.64 (0.73)	0.018*	0.44
“Think about taking life”	1.25 (0.43)	1.05 (0.16)	1.38 (0.51)	0.034*	0.41	1.07 (0.17)	1.35 (0.50)	0.046*	0.33
SI composite	1.38 (0.61)	1.07 (0.17)	1.60 (0.71)	0.015*	0.48	1.98 (0.19)	1.54 (0.70)	0.025*	0.38
EMA SI worst‐point severity									
“Life not worth living”	3.44 (1.83)	2.30 (1.59)	4.25 (1.56)	0.002*	0.62	2.65 (1.87)	3.87 (1.69)	0.048*	0.34
“More reasons to die than to live”	3.06 (1.83)	2.15 (1.31)	3.71 (1.88)	0.005*	0.47	2.41 (1.54)	3.42 (1.89)	0.062	0.27
“Want to die”	3.44 (1.93)	2.10 (1.25)	4.39 (1.77)	<0.001**	0.72	2.24 (1.44)	4.10 (1.87)	0.003*	0.52
“Think about taking life”	2.77 (1.79)	1.70 (0.98)	3.54 (1.86)	<0.001**	0.59	1.76 (1.03)	3.32 (1.89)	0.003*	0.46
SI composite	2.90 (1.59)	1.91 (1.01)	3.61 (1.56)	<0.001**	0.62	2.09 (1.13)	3.35 (1.64)	0.005*	0.41
EMA compliance	51.13 (13.23)	55.20 (10.03)	50.43 (13.50)	0.188	0.19	54.41 (8.87)	51.32 (13.83)	0.411	0.12

*Note*: SI composites were average scores across the four suicidal ideation items (1 = *Not at all;* 7 = *Completely*). Reported *p*‐values were adjusted using the Benjamini‐Hochberg Procedure to control the false discovery rate.

Abbreviations: *d*, Cohen's *d*; EMA, ecological momentary assessment; SI, suicidal ideation.

*p < 0.05. **p < 0.001.

When examining the degree of agreement in SI endorsements between the follow‐up survey and interview, 65 (89.04%) out of 73 adolescents who completed follow‐up assessments provided consistent reporting across both; 27 (36.99%) consistently endorsed SI, and 38 (52.05%) consistently denied any SI during the EMA period. However, 8 adolescents (10.96%) showed discrepant reporting; 5 (6.85%) endorsed SI on the interview, but not on the survey whereas the other 3 (4.11%) endorsed SI on the survey, but not on the interview.

### Discrepancies between EMA and retrospective measures

We examined discrepancies in SI endorsement between EMA and retrospective, follow‐up measures (survey, interview). The rates of discrepancies are presented in Table 3.^3^ On the follow‐up survey, over half (58.33%, *n* = 28) of adolescents who endorsed SI on EMA consistently endorsed having experienced SI during the EMA period (“EMA SI+/FU Survey SI+”). However, 20 (41.67%) retrospectively denied any SI during the EMA period (“EMA SI+/FU Survey SI−”). Across individual SI items, the rates of retrospective denial ranged from 39.02% to 50.00%.

**TABLE 3:** Frequency of retrospective denial of momentary suicidal ideation.

	EMA SI+	EMA SI+/FU survey SI−	EMA SI+/FU interview SI−
“Life is/was not worth living for me”	41	16 (39.02%)	15 (36.59%)
“There are/were more reasons to die than to live for me”	36	18 (50.00%)	21 (58.33%)
“I want(ed) to die”	39	16 (41.03%)	15 (38.46%)
“I think/thought about taking my life”	32	13 (40.63%)	15 (46.88%)
SI composite	48	20 (41.67%)	17 (35.42%)

*Note*: SI composites were average scores across the four suicidal ideation items (1 = *Not at all;* 7 = *Completely*).

Abbreviations: EMA, ecological momentary assessment; SI, suicidal ideation.

In the follow‐up interview, 64.58% (*n* = 31) of adolescents who endorsed SI via EMA consistently reported having experienced SI during the EMA period (“EMA SI+/FU Interview SI+”); 17 adolescents (35.42%) made discrepant reports, endorsing SI on EMA, but not in the follow‐up interview (“EMA SI+/FU Interview SI−”). The rate of discrepancies ranged from 36.59% to 58.33% across individual SI items.

### Associations between EMA‐retrospective measures discrepancies and EMA SI variables

We compared adolescents with and without discrepant reports across EMA and retrospective measures based on EMA SI frequency and severity and EMA compliance (see Table 2). Compared to the EMA SI+/FU Survey SI+ group, the EMA SI+/FU Survey SI− group showed fewer occurrences of SI and lower SI severity based on mean and worst‐point SI ratings reported during the EMA period.^4^ Similarly, compared to the EMA SI+/FU Interview SI+ group, the EMA SI+/FU Interview SI− group demonstrated fewer occurrences of SI and lower SI severity based on mean and worst‐point SI ratings reported during the EMA period. When comparing EMA compliance, no significant differences were found between the EMA SI+/Follow‐Up Survey SI+ and SI− groups, nor between the EMA SI+/Follow‐Up Interview SI+ and SI− groups.

### Demographic and clinical correlates of EMA‐retrospective measures discrepancies

Table 1 presents comparisons of adolescents who retrospectively denied SI during the EMA period and those who did not based on demographic and clinical characteristics. There were no significant differences between the EMA SI+/FU Survey SI− and EMA SI+/FU Survey SI+ groups based on biological sex, gender, race, ethnicity, sexual orientation, and age, *ps* = 0.24–1.00. The EMA SI+/FU Interview SI+ and EMA SI+/FU Interview SI− groups also did not show demographic differences, *ps* = 0.54–1.00.

The two groups based on the FU survey (EMA SI+/FU Survey SI− vs. EMA SI+/FU Survey SI+) significantly differed on several clinical variables, such that adolescents without discrepancies demonstrated a higher likelihood of lifetime SI and suicide attempt than those with discrepancies, *ps* = 0.001–0.037. At baseline, the former group was also more likely to receive mental health treatment and scored higher on SI severity in the past 2 weeks (SIQ), depression severity in the past week (QIDS), and hopelessness (HSC), *ps* = 0.001–0.032. When comparing the groups based on the FU interview (EMA SI+/FU Interview SI+ vs. EMA SI+/FU Interview SI−), adolescents without discrepancies also showed a higher likelihood of lifetime SI and greater SI severity in the past 2 weeks (SIQ), depression severity in the past week (QIDS), and hopelessness (HSC) compared to those with discrepancies, *ps* = 0.004. However, the groups did not vary based on the likelihood of a lifetime suicide attempt or mental health treatment‐seeking status at baseline, *ps* = 0.086–0.088.

For both sets of comparisons (EMA SI+/FU Survey SI+ vs. EMA SI+/FU Survey SI−; EMA SI+/FU Interview SI+ vs. EMA SI+/FU Interview SI−), no significant group differences were found on the change in mental health treatment status during EMA, anxiety severity in the past 3 months (SCARED), rumination (RRS), worry (PSWQ‐C), mental health stigma (STIG‐9), and self‐reflection and insight (SRIS‐Y), *ps* = 0.056–1.00. Results also indicated no group differences on adolescents' perceived accuracy of their responses on follow‐up measures, with all adolescents reporting that they provided accurate responses, *ps* = 0.10–0.68.

## CONCLUSIONS

The detection of suicidal thinking in adolescents is a key component of suicide prevention, yet a growing body of research suggests different assessment methods yield discrepant reports. This study is the first to examine what accounts for discrepancies in SI reported by adolescents from the community in real‐time via EMA versus retrospectively via interview and self‐report survey. We found that a sizable subgroup of adolescents (35%–42%) who endorse some level of SI via EMA over the course of a 2 week‐period subsequently denied it altogether when assessed retrospectively at the EMA period's end. Our study, including retrospective comparison points of both an interview and a self‐report survey, suggests both modes of retrospective assessment yield comparable levels of under‐reported SI as compared with EMA. Adolescents were more likely to provide discrepant reports if they experienced fewer or less severe SI during the EMA period, and they presented with less clinical severity based on prior history of SI and suicide attempt and psychopathology.

The present finding that EMA captures SI that is not captured by traditional retrospective assessments among community‐based adolescents contributes to a growing body of work demonstrating that retrospective measures capture less frequent or severe SI than real‐time reports in both adolescents (Czyz et al., 2018; Esposito et al., 2022) and adults (Forkmann et al., 2018; Gratch, Choo, et al., 2021; Torous et al., 2015). For instance, both Czyz et al. (2018) and Esposito et al. (2022) assessed adolescent psychiatric samples and demonstrated that 36%–43% of adolescents endorsing SI via daily diary/EMA later denied it retrospectively; the present study suggests similarly high levels of discrepancy (35%–42%) exist in a community sample of adolescents representing a wider range of STB severity and treatment engagement. Some have hypothesized that these discrepancies are partially attributable to differences in administration method, that is, with disclosure to an *interviewer* in a retrospective interview resulting in under‐reported SI compared to self‐report surveys completed via a phone app during EMA (e.g., Spears et al., 2022). Notably, our study, detecting discrepancies between EMA and the retrospective interview *and* EMA and the retrospective self‐report survey, suggest that differing timescales—that is, asking participants to report SI over a past period as opposed to right now—is a key contributor to observed discrepancies.

At the same time, it is somewhat surprising that the current study found a similar rate of discrepancy between EMA and retrospective measures compared to previous studies (Czyz et al., 2018; Esposito et al., 2022), despite employing the shorter EMA duration (i.e., 2 weeks vs. 28–30 days). Given that recall accuracy tends to decrease over longer time intervals (e.g., Bradburn et al., 1987; Schwarz & Oyserman, 2001), one might expect discrepancy rates to be lower when participants recall over the past 2 weeks as opposed to over the past month. At the same time, it is of note that the prior studies focused on high‐risk suicidal adolescents recently discharged from acute care (e.g., emergency department, inpatient). Given our finding that higher clinical acuity is associated with a lower likelihood of retrospective denial of SI, it is possible that higher‐acuity samples (e.g., psychiatric inpatients) may demonstrate more accurate retrospective recall than community samples. The combination of these factors may explain the high degree of correspondence in the retrospective discrepancy rates observed across the current and prior studies despite their methodological differences (e.g., EMA duration, sample characteristics).

Theory and empirical research in cognitive psychology inform an explanation as to why the difference in timescales may drive discrepancies between EMA and retrospective assessments of SI. Consistent with Fredrickson and Kahneman's (1993) “peak‐end rule,” posing that our recall of an experience is most influenced by the peak and end points of that experience, our findings suggest that the worst point during EMA is important for adolescents' retrospective formulations; worst‐point SI severity across almost all EMA items was significantly higher for those whose EMA‐retrospective survey and EMA‐retrospective interview reports were concordant versus those whose were discrepant. This is consistent with Gratch, Choo, et al. (2021) finding that worst‐point SI during EMA correlated with retrospective reports among depressed adults. Similarly, Kiefer et al. (2024) found that peak post‐traumatic stress disorder (PTSD) symptoms as reported by women three times daily over the course of a month were more predictive of retrospectively reported PTSD symptoms than were the averages. At the same time, we were unable to test the end‐point hypothesis due to limited variability in end‐point SI ratings within the current sample. As a result, conclusions regarding the end‐point hypothesis remain inconclusive. Future studies using samples with higher clinical acuity (vs. community‐based samples) may be necessary to adequately evaluate this question. Nonetheless, the current findings suggest that adolescents' recollections of SI may be especially shaped by their worst‐point or peak experiences.

The fact that worst‐point SI during EMA was associated with greater likelihood of reporting SI retrospectively may also be attributable to overall differences in clinical severity between those whose reports were concordant versus discrepant. Consistent with previous research (e.g., Gratch, Choo, et al., 2021), we found that adolescents who denied SI retrospectively (i.e., demonstrating EMA‐retrospective survey and/or EMA‐retrospective interview discrepancies) reported fewer instances of SI and less severe mean and peak SI during EMA, lower likelihood of lifetime SI, and less severe SI, hopelessness, and depressive symptomatology at baseline than those whose reports were concordant. Those who endorsed SI during EMA and the retrospective survey also had higher hopelessness, higher likelihood of lifetime suicide attempt, and mental health treatment history at baseline.

Other factors hypothesized to contribute to discrepancies across assessment methods did not emerge as relevant in this study, including demographic characteristics, mental health stigma, self‐reflection, insight, and rumination. Furthermore, when reporting in the exit survey how accurate adolescents *believed* their responses to retrospective assessments to be, this perceived accuracy did not map onto actual, observed discrepancies, suggesting that this pattern of under‐reporting is likely outside of conscious awareness. Taken together, these findings suggest that discrepancies are probably not primarily attributable to lack of insight or self‐reflection, concerns about disclosure, or intentional evasiveness. Rather, retrospectively under‐reported SI appears to be, at least in part, a reflection of the level of clinical severity of the respondent, with adolescents experiencing more intense and frequent suicidal distress and related psychopathology (hopelessness, suicidal behavior, depressive symptomatology) being more likely to report SI consistently across methods.

While it is somewhat reassuring that those in more distress are likely to report SI retrospectively as well as in real‐time, the present findings and extant research raise critical implications for clinicians; emerging findings repeatedly suggest the presence of a subgroup of suicidal individuals, including adolescents, whose suicidal thinking may be more difficult to capture using retrospective measures, which are typically relied upon in clinical settings. This is especially notable for individuals with the recent onset of SI, whose SI, even at lower levels, would be important to capture to intervene earlier in the suicide trajectory. The current study contributes to a growing consensus that EMA may have greater utility in capturing less severe, early suicidal thoughts; detecting it in adolescence could be an important part of the suicide prevention efforts.

Real‐time monitoring could also be a clinically useful tool for adolescents with low levels of SI who are already engaged in treatment and whose SI may not be elicited via a standard retrospective question asked in session. This could be particularly apt in treatments where patients are already engaging in some form of self‐monitoring between sessions (e.g., cognitive behavioral therapy; Cohen et al., 2013). Such an approach may better elucidate the nature and extent of suicidal thinking in daily life and enhance the clinician's ability to address it in sessions. Though more research is needed to tease apart potential drawbacks and benefits of frequent assessment of STBs in a treatment context (Law et al., 2015), preliminary research suggests high feasibility and acceptability of EMA in high‐risk adolescent samples, including no evidence of iatrogenic effects (Czyz et al., 2018; Glenn et al., 2020; Love et al., 2024; Shin et al., 2024).

Findings of the present study should be considered in light of several limitations. First, our sample size was relatively small, and several demographic subgroups included very few participants. As a result, null findings may reflect limited statistical power rather than true absence of effects and should be interpreted with caution. At the same time, we capture a diverse cross‐section of suicidal adolescents from the community, and findings clearly replicate patterns observed in previous research. Second, although our adolescent sample was relatively diverse in race, ethnicity, and sexual orientation, we did not assess socioeconomic status. On one hand, requiring smartphone access may have underrepresented adolescents from lower socioeconomic backgrounds in the current sample. At the same time, this concern is mitigated by data showing that 95% of U.S. adolescents report having access to a smartphone (Pew Research Center, 2024). Third, because we do not have a long‐term follow‐up, we cannot say that the lower clinical severity associated with greater discrepancies is actually associated with reduced future risk. Indeed, previous research suggests that passive ideation is just as likely to lead to suicidal behavior as active ideation (Liu et al., 2020). In this way, it is not completely clear what the undetected SI means in terms of future risk for the subgroup of youth endorsing SI, who appear less clinically severe. Future research would benefit from examining prospective trajectories of STBs among adolescents whose SI is detected only through real‐time monitoring and not through retrospective assessments.

There are additional avenues to extend the current findings. First, although EMA compliance was not associated with retrospective denial in the current sample, it remains possible that a minimum threshold of compliance is necessary for EMA to provide greater sensitivity in detecting SI compared to retrospective assessments. Clarifying this threshold would inform future clinical applications of EMA in suicide risk assessment and detection. Another future direction is to examine the duration of suicidal thoughts and its potential impact on the retrospective underreporting of such thoughts. While the current findings suggest that less frequent or less intense SI tends to be underreported to a greater extent, the duration of SI may also play a role: fleeting or transient thoughts may be more difficult to recall than longer‐lasting or persistent thoughts. Relatedly, emerging evidence indicates that different types of SI vary in their time course (e.g., elevated suicidal intent persisting for a shorter period than elevated suicidal desire; Coppersmith et al., 2023). Investigating whether and how these temporal characteristics of SI may factor into the accuracy of retrospective reporting would provide further insights into refining suicide risk assessment methods to more accurately capture the full spectrum of suicidal thoughts.

To conclude, the present study contributes to a growing understanding of the complexity of assessing STBs based on self‐report. Our findings highlight the potential strengths of real‐time monitoring as a key component of suicide prevention for adolescents.

## AUTHOR CONTRIBUTIONS

**Ki Eun Shin**: Conceptualization; formal analysis; methodology; supervision; writing—original draft. **Ilana Gratch**: Conceptualization; methodology; writing—original draft. **Christine B. Cha**: Conceptualization; funding acquisition; methodology; resources; supervision; writing—review and editing.

## CONFLICT OF INTEREST STATEMENT

The authors declare no conflicts of interest.

## ETHICAL CONSIDERATIONS

Informed assent was obtained from all adolescent participants and informed consent from their parents or legal guardians. The study protocol was reviewed and approved by the Institutional Review Board at Teachers College, Columbia University (approval date: April 21, 2021; protocol number: 21–279).

## Data Availability

The data that support the findings will be made publicly available in the Open Science Framework once the study team's other manuscripts based on the data set are accepted for publication.

## ENDNOTES

^1^
Although we categorized “*I wanted to die*” as an indicator of active SI in line with the prior EMA item validation study (Forkmann et al., 2018), we recognize that this item may conceptually overlap with the operationalization of passive SI in other suicide risk measures (e.g., “*wish to be dead*” in the Columbia–Suicide Severity Rating Scale; Posner et al., 2011). The prior validation study demonstrated that passive and active SI, based on the same EMA items used in the current study, emerged as distinct latent constructs but were highly correlated (*r* = 0.75). While our analyses examined SI at the item and composite levels without aggregating items into passive and active SI categories, we acknowledge this potential overlap as a consideration for interpreting the results.

^2^
In case participants endorsed active SI (>1 = *Not at all*), they were prompted to rate their suicidal intent, or the likelihood of acting on suicidal thoughts in the next 24 h (1 = *Not at all likely*; 7 = *Very likely*), as well as ability to keep themselves safe (1 = *Definitely can keep myself safe*; 7 = *Definitely cannot keep myself safe*). Similar items were used for safety monitoring in a prior EMA survey involving suicidal adolescents (Glenn et al., 2020).

^3^
Two adolescents did not endorse any SI during EMA, but retrospectively reported SI on the follow‐up survey. Similarly, one adolescent did not endorse any SI during EMA, but retrospectively reported SI in the follow‐up interview. While we note these discrepancies, those participants were not included in group comparisons due to the prohibitively small group sizes.

^4^
The end‐point ratings across the four SI items and the SI composite demonstrated limited variability in the EMA SI+/FU Survey SI− group (*M* = 1.00–1.01, SD = 0.04–0.43) and the EMA SI+/FU Interview SI− group (*M* = 1.00–1.01, SD = 0.00–0.06). Due to concerns about restricted range and limited statistical power, we did not pursue group comparisons on the end‐point SI ratings.
